# The Influence of Novel, Biocompatible, and Bioresorbable Poly(3-hydroxyoctanoate) Dressings on Wound Healing in Mice

**DOI:** 10.3390/ijms232416159

**Published:** 2022-12-18

**Authors:** Martyna Seta, Katarzyna Haraźna, Kaja Kasarełło, Daria Solarz-Keller, Agnieszka Cudnoch-Jędrzejewska, Tomasz Witko, Zenon Rajfur, Maciej Guzik

**Affiliations:** 1 Chair and Department of Experimental and Clinical Physiology, Medical University of Warsaw, Banacha 1b, 02-091 Warsaw, Poland; 2 Jerzy Haber Institute of Catalysis, Surface Chemistry Polish Academy of Sciences, Niezapominajek 8, 30-239 Krakow, Poland; 3 Faculty of Physics, Astronomy and Applied Computer Science, Jagiellonian University, Lojasiewicza 11, 30-348 Krakow, Poland

**Keywords:** wound healing, poly(3-hydroxyoctanoate), P(3HO), polyhydroxyalkanoates, porous patches, dressing materials, bioresorption, angiogenesis, CD68* macrophages

## Abstract

The human body’s natural protective barrier, the skin, is exposed daily to minor or major mechanical trauma, which can compromise its integrity. Therefore, the search for new dressing materials that can offer new functionalisation is fully justified. In this work, the development of two new types of dressings based on poly(3-hydroxyoctanoate) (P(3HO)) is presented. One of the groups was supplemented with conjugates of an anti-inflammatory substance (diclofenac) that was covalently linked to oligomers of hydroxycarboxylic acids (Oli-dicP(3HO)). The novel dressings were prepared using the solvent casting/particulate leaching technique. To our knowledge, this is the first paper in which P(3HO)-based dressings were used in mice wound treatment. The results of our research confirm that dressings based on P(3HO) are safe, do not induce an inflammatory response, reduce the expression of pro-inflammatory cytokines, provide adequate wound moisture, support angiogenesis, and, thanks to their hydrophobic characteristics, provide an ideal protective barrier. Newly designed dressings containing Oli-dicP(3HO) can promote tissue regeneration by partially reducing the inflammation at the injury site. To conclude, the presented materials might be potential candidates as excellent dressings for wound treatment.

## 1. Introduction

The skin, the human body’s largest organ, is a barrier that separates the human body from the external environment. During injury, the integrity of the skin is disrupted and the immune response to inflammation is impaired. This finally leads to the formation of wounds [1]. Despite the many efforts, research conducted, and implementation work carried out worldwide, no ideal universal wound dressing has yet been developed. Due to the many studies conducted and the considerable demand for dressing materials, the market for wound care products is expected to be valued at 15–22 billion dollars in 2024 [2]. The primary purpose of wound dressings is to protect the wound from the external environment. Traditional wound dressings, i.e., gauze, natural or synthetic bandages, and cotton, lower the wound area’s pH and synthesise collagen at the wound site, which ultimately promotes the reepithelialisation process. In addition, such dressings provide a moist environment within the wound and allow the absorption of fluids [3,4]. There are more than 3000 different types of wound dressings available on the market worldwide [4]. Many studies describe the use of polymers in the construction of natural and synthetic dressing materials [5]. Natural polymers, independent of the source (i.e., animal, vegetable, or microbial), have many advantages, including biocompatibility, biodegradability, and bioactivity, allowing the replacement of natural extracellular matrix (ECM) components [6].

Modern wound dressings require the reproduction of the natural environment, enabling cell adhesion and proliferation at a precise location. These materials should have a porous structure. Using dressings with high porosity will allow a more efficient cell migration as well as proliferation and effective transport of nutrients, active substances, oxygen, and metabolites to and from the regenerated site [7]. The most common techniques used to produce porous wound dressings include the solvent casting/particulate leaching (SCPL) technique, electrospinning, 3D printing, gas foaming, supercritical fluid processing, and stereolithography [8]. The SCPL is one of the most straightforward techniques, not requiring complicated and expensive equipment or raw materials. It allows a material to be obtained that is characterised by well-defined parameters such as size, shape, and number of pores [9]. The possibility of applying polymeric porous dressings, made by the SCPL technique, in skin regeneration has been previously evaluated in numerous in vivo studies. For this purpose, both synthetic and natural polymers were used, i.e., poly(lactide-*co*-glycolide) (PLGA), polyurethane (PUR), chitosan, and nanocellulose [7,10,11,12,13]. Moreover, the SCPL technique allows it to create scaffolds that supply active substances, drugs, or proteins at a specific place [7,11,14,15,16].

According to general knowledge, modern wound dressings should be characterised by various features that include appropriate gas permeability, elasticity, lack of toxicity, biocompatibility, and biodegradability. However, it remains a significant challenge to obtain dressings characterised by the features mentioned above, which at the same time could meet several conditions, i.e., (1) being easy to change/remove from the wound site; (2) protecting the wound from infection and microorganisms; (3) controlling moisture within the wound site; (4) reducing the necrosis of the wound surface; (5) and alleviating patient pain. For this purpose, research is still ongoing to find even better equivalents [4]. During the wound-healing process, infection or prolonged inflammation can slow down the regeneration of the damaged area by inhibiting its contraction [17]. To improve the wound regeneration process, recent research has focused on developing dressings that can accelerate the regeneration process by eliminating infection or reducing the intensity of inflammation [3,4]. This is possible by supplementing the dressings with appropriate drugs or bioactive molecules. Rancan et al. investigated the feasibility of using polymeric matrices made of polyvinylpyrrolidone (PVP) containing an antibiotic, namely ciprofloxacin. In vivo studies have shown that supplementing the patches with the antimicrobial drug positively affected the wound healing process. These are promising systems for treating or curing wounds infected by bacterial infection [18].

Pain is often accompanied by skin wounds. It is associated with dressing changes and cleansing processes of wound sites and can be caused by exacerbated inflammation within the damage. Non-steroidal anti-inflammatory drugs (NSAIDs) and steroids are the most commonly used anti-inflammatory drugs in the construction of transdermal wound dressings [19]. Diclofenac, a representative NSAID, has a broad spectrum of applications, from the treatment of rheumatologic diseases to the relief of pain after extensive surgical interventions. It owes its action to its ability to reduce the levels of cyclooxygenases, which ultimately leads to a reduction in the levels of prostaglandins produced, which cause inflammation and pain [20,21]. The use of diclofenac in the construction of modern wound dressings has been described in the literature. Postolovic et al. demonstrated the applicability of dressings prepared from κ-carrageenan, alginate, and poloxamer polymers incorporated with diclofenac in the treating of chronic burn wounds. Histopathological analysis of burned skin treated with the prepared dressings showed the regeneration of damaged tissue, which was substantiated by reduced inflammatory cell infiltration [20].

The use of biocompatible polymers, i.e., polyhydroxyalkanoates (PHAs), in the construction of dressing materials may be an excellent approach to improve patient comfort. This is because there is no need to change the dressing as these polymers are fully resorbable [22]. Additionally, they are biocompatible and biodegradable [23,24]. Therefore, it is an interesting material for many medical applications, from the construction of drug carriers to the creation of dressing materials or scaffolds for bone tissue regeneration processes [25,26,27,28,29,30,31,32,33].

Poly(3-hydroxyoctanoate) (P(3HO)), a representative medium chain length polyhydroxyalkanoate (mcl-PHA), is an elastomer with a low degree of crystallinity and tensile strength, a low melting point, and a high elongation at break [32]. The average value of Young’s modulus of the dermis for women aged 29–66 years taken from the breast and abdomen is estimated at 770 Pa [34]. Accordingly, materials made using P(3HO) can be excellent candidates for skin regeneration applications. This is related to the fact that Young’s modulus for materials prepared from P(3HO) is 12.6–25.4 MPa [24]. Another reason for using P(3HO) in the construction of modern dressing materials is the lack of cytotoxicity of the degradation of this polymer [23,24]. Moreover, the degradation products of P(3HO) do not cause inflammation in contact with mammalian tissues, which is related, among other things, to the weak acidity of (R)-3-hydroxycarboxylic acids (pKa = 4.84) [23,24,25]. A further argument favouring the applicability of P(3HO) is an analysis of the wound healing process conducted for a mouse embryonic fibroblast model (MEF3T3). This study showed a similar rate of wound overgrowth for P(3HO) and the control material (glass) [26]. All the data mentioned above show that P(3HO) is a promising candidate for wound dressing constructions.

The presented study aimed to assess the biomedical potential of created polymeric porous patches exhibiting anti-inflammatory properties. For the production of polymeric patches, a representative of medium-chain-length polyhydroxyalkanoates—pol(3-hydroxyoctanoate) P(3HO)—was used. Diclofenac, which was attached to 3-hydroxycarboxylic acids by a covalent bond, was applied as the substance showing anti-inflammatory activity. Afterwards, porous dressing materials were prepared using the solvent casting/particulate leaching (SCPL) technique. To the best of our knowledge, no studies have been conducted on P(3HO) polymer for this particular purpose. The paper presents a method of selecting the concentration of active substance conjugates used to prepare bioactive dressings (cytotoxicity test). Then, the surface and mechanical properties of the prepared materials were evaluated, as well as the behaviour of mouse fibroblast cells on them. Finally, the possibilities of using the prepared dressings in wound healing in vivo were investigated. This process was assessed using immunohistochemistry and changes in mRNA expression and by estimating levels of selected interleukins.

## 2. Results and Discussion

### 2.1. The Physicochemical Characterisation of Diclofenac Conjugates

Tailor-made surface functionalisation of biopolymers could be a very interesting approach for applications in the preparation of novel dressings. The undoubted advantage of drug–polymer conjugates can be the controlled release of active substances directly into the injury site [35,36]. Therefore, we have integrated diclofenac-modified oligomers in the structure of P(3HO) derived materials to create a novel anti-inflammatory material for wound healing applications.

**Figure 1:**
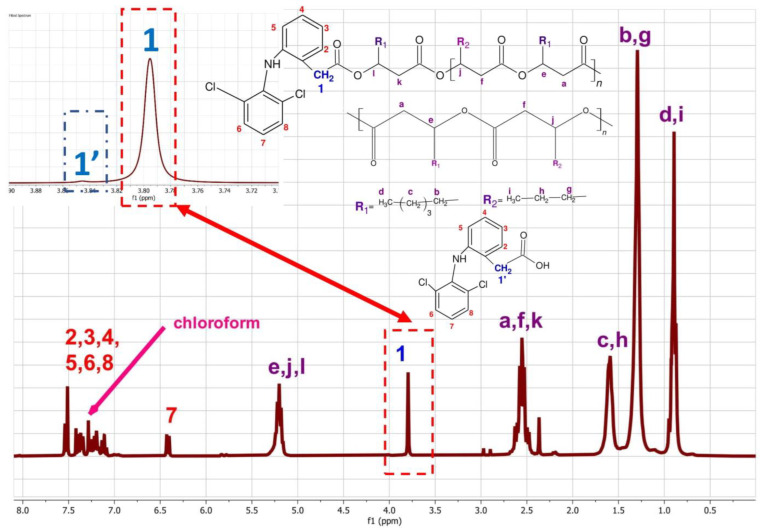
1H NMR of oli-dic-P(3HO) analyses, where (1) denotes the CH2 groups from DIC attached to oligomers and (1′) denotes groups from free DIC. The pictures also show labels of the assigned hydrogen atoms and the structural formulas of the compounds of the reaction mixture.

**Figure A1:**
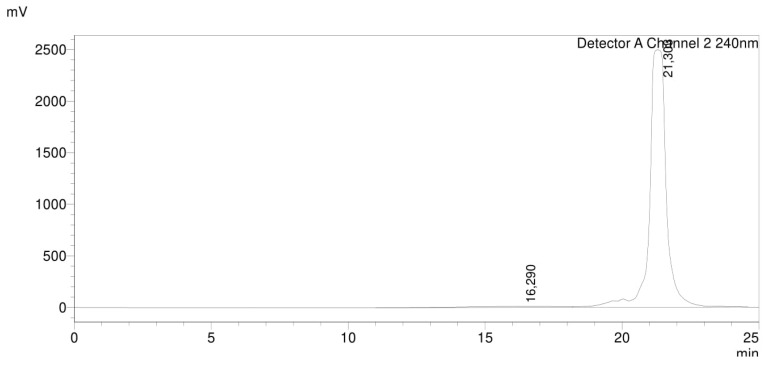
GPC profile of oli-dicP(3HO).

**Figure A2:**
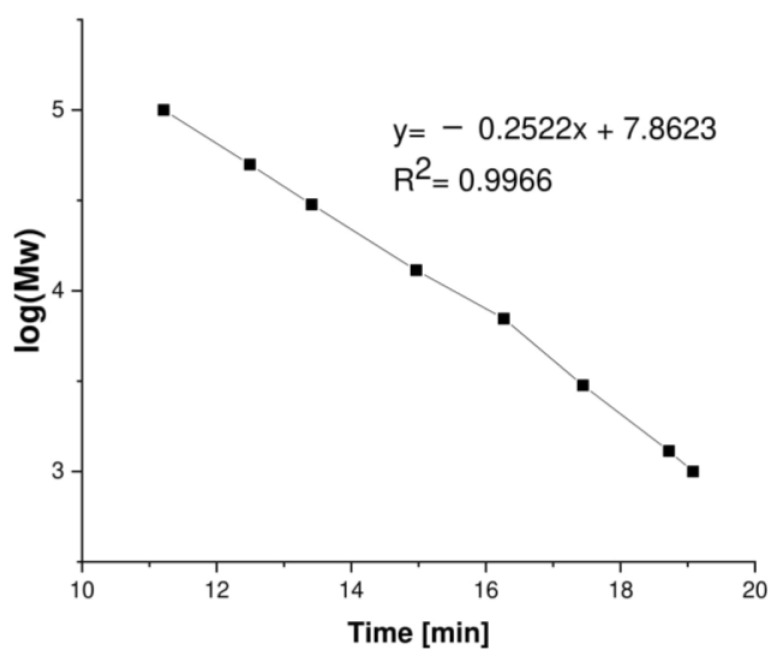
Calibration curve of GPC prepared using polystyrene (PS) standards.

The analysis of ^1^H NMR spectra showed that the obtained reaction mixture consists of three substances—3-hydroxyfatty acid oligomers modified with diclofenac, unmodified oligomers, and a small amount of unreacted drug (Figure 1). As we have shown in previous work, it is possible to distinguish signals originating from diclofenac chemically bound to oligomers via an ester bond (Figure 1, signal 1) and the remaining unreacted compound (Figure 1, signal 1′) [31]. The signal at a shift of 3.78 ppm corresponds to the hydrogen of the methylene group, which is adjacent to the ester group connecting the drug molecule to the 3-hydroxyalkanoate oligomers. In turn, the signal at a shift of 3.85 ppm corresponds to the hydrogens of the free non-bonded diclofenac methylene group. Calculations using Equation (1) showed that 1 g of the resulting mixture contains only 3.7 mg of unreacted diclofenac, while 226 mg of the drug is covalently bound to the oligomers. Moreover, the use of a catalyst, which is also a factor in the decomposition of the polymer, significantly reduces the oligomer population’s molecular weight and dispersity (Table 1, Figure A1 and Figure A2).

### 2.2. Description of Surface and Mechanical Properties of Materials

**Figure 2:**
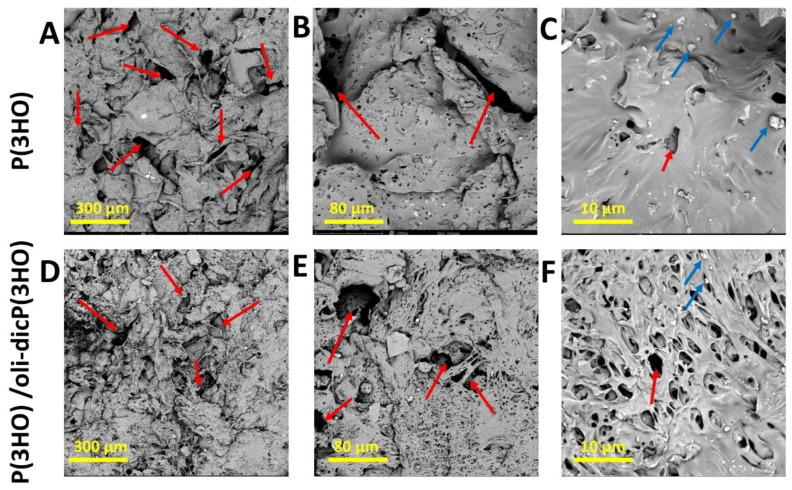
SEM image (**A**–**C**) of P(3HO) foams; (**D**–**F**) of P(3HO)/oli-dicP(3HO). View (**A**,**D**) ×220; (**B**,**E**) ×1000; (**C**,**F**) ×5000. In the figure, red arrows mark the pores, and blue arrows mark the grains of unleached salt.

In our work, three-dimensional porous foams were prepared using the SCPL technique. An unquestionable advantage of this technique is the possibility of selecting the pore size, which depends on the porogen’s grain size. The macrostructures of the obtained foams are shown in Figure 2A,B,D,E. It can be seen that the use of porogen with grain sizes between 100 and 300 µm results in materials with pores < 200 µm. Furthermore, Figure 2C,F show that the foams also have smaller pores with a diameter < 5 µm. Smaller porogen grains are responsible for the forming of the smaller-diameter pores. They may be formed during one of the steps used in the preparation of the materials (i.e., mixing of porogen grains with the polymer solution). Further, the microphotographs show a small amount of unleached porogen (grains < 5 µm).

**Figure 3:**
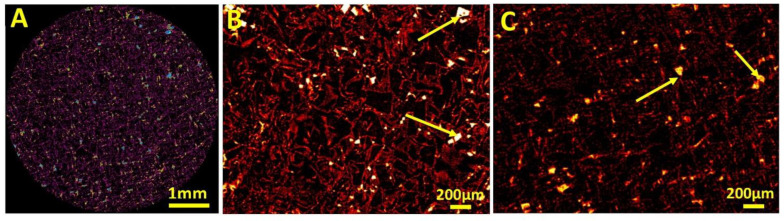
(**A**–**C**) The reconstructed cross-section of P(3HO) patch, obtained through the µ-CT method. Yellow arrows indicate porogen grains present in the prepared material.

The microcomputed tomography (μ-CT) results clearly show the porous structure of the prepared materials (Figure 3A–C). The prepared patches based on P(3HO) are characterised by high porosity, which was 96%. The patch visualised in the included photographs was prepared using a method combining porogen leaching and solvent evaporation. Therefore, during the planning and implementation stage of the methodology, there was already a risk that it would not be possible to completely wash out the grains of the applied porogen—sodium chloride. The hypothesis was confirmed, as visualised in photographs (Figure 3B,C) of a cross-section of the obtained patch. In the case of the materials we have constructed, all the above-given information from the literature and provided by the obtained results may be highly desirable in patch construction. The degradation of the polymeric material during the overgrowth stage can result in the release of diclofenac-modified oligomers directly into the wound area. This will inhibit the inflammation occurrence at the regenerated site. In addition, degradation of the polymer itself will lead to the release of 3-hydroxyfatty acids, which will further nourish the wound [31]. Furthermore, small pores can allow excellent diffusion of nutrients deep into the wound and metabolic products to the outside of the place of injury [37]. The residual unleached porogen (sodium chloride), confirmed by the SEM and µ-CT analysis, should not interfere with skin regeneration.

Many parameters have to be considered during the design of scaffolds for skin tissue regeneration, i.e., the physicochemical properties as well as the number, size, and shape of the pores. Through the pores, cells adhere to the surface, remove metabolites, and absorb nutrients [7]. Different literature data give divergent information regarding the optimal pore size of scaffolds for skin regeneration. Yannass et al. showed that the ideal pore size in scaffolds designed for skin tissue regeneration should be 50–200 µm [38]. Choi et al. showed that proliferation, carried out for 14 days on a human dermal fibroblast cell line on scaffolds with a larger pore size (<580 µm), was more efficient compared to scaffolds containing smaller-diameter pores (<435 µm) [39]. In addition, scaffolds with porosity higher than 90% have been shown to promote more efficient proliferation and ECM reconstruction than scaffolds with porosity around 70% [40]. Pore size is also very important in the biodegradation of dressing materials. As demonstrated by Van Tienen et al. the presence of micropores can promote the onset of biodegradation of the dressing material before the stage of overgrowth by cells of the newly formed tissue is completed [41].

**Figure 4:**
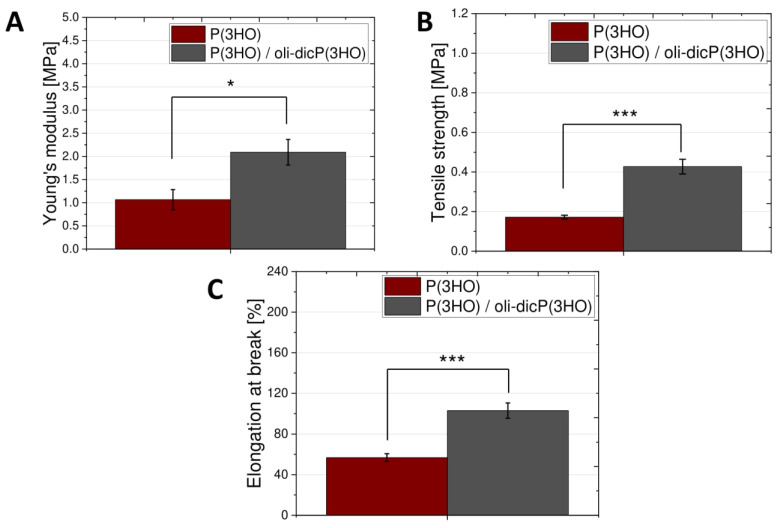
The Young’s modulus (**A**), tensile strength (**B**), and elongation at break (**C**) of prepared foams. The results are statistically significant, where: * *p* < 0.05, *** *p* < 0.001.

An additional requirement for a modern wound dressing is its appropriate mechanical properties, which provide adequate stability, flexibility, softness, and elasticity to cover the entire wound surface during healing [42]. The effect of the addition of diclofenac-modified oligomers on the mechanical properties, i.e., Young’s modulus (Figure 4A), tensile strength (Figure 4B), and elongation at break (Figure 4C) of the prepared foams were evaluated in this work. The addition of modified oligomers caused an increase in all the parameters mentioned above compared to foams made of P(3HO) polymer. Young’s modulus, tensile strength, and elongation at break for P(3HO)/oli-dicP(3HO) and P(3HO) foams were: 2.09 ± 0.28 MPa, 0.43 ± 0.04 MPa, 102.95 ± 7.53% and 1.07 ± 0.22 MPa, 0.17 ± 0.01 MPa, 51.98 ± 3.83%, respectively.

Ruppert et al. showed that the tensile strength, elongation at break, and Young’s modulus for human skin and scar are: 1.75 ± 1.0 MPa, 248 ± 49%, 1.40 ± 0.50 MPa, and 1.99 ± 1.48 MPa, 144 ± 38%, 2.96 ± 1.19 MPa, respectively [7]. The lower value of Young’s modulus obtained in our study for foams made of P(3HO), which was 1.07 ± 0.22 MPa, is very desirable. This is because a lower Young’s modulus will allow for greater flexibility of the scaffolds at the location used [7]. The values of tensile strength and elongation at break of the foams obtained in the following work are too low and for P(3HO) and P(3HO)/oli-dicP(3HO) foams amount to 0.17 ± 0.01 MPa, 51.98 ± 3.83% and 0.43 ± 0.04 MPa, 102.95 ± 7.53%, respectively. This problem can be eliminated using biocompatible plasticisers in future designs [11]. Such a plasticiser could be unmodified oligomers of 3-hydroxy acids. Lukasiewicz et al. showed that elongation at break increases with the amount of 3-hydroxy acid oligomers. At the same time, a negative phenomenon was observed concerning the decrease in tensile strength with an increase in the content of 3-hydroxy acids oligomers [43]. Moreover, the average Young’s modulus value of the dermis for women aged 29–66 years, taken from the breast and abdomen, was 770 Pa [34]. Therefore, it can be concluded that materials made of modified polyhydroxyoctanoate will be appropriate for skin regeneration.

### 2.3. Cytotoxicity Assessment of Diclofenac-Modified Conjugates (Oli-dicP(3HO))

**Figure 5:**
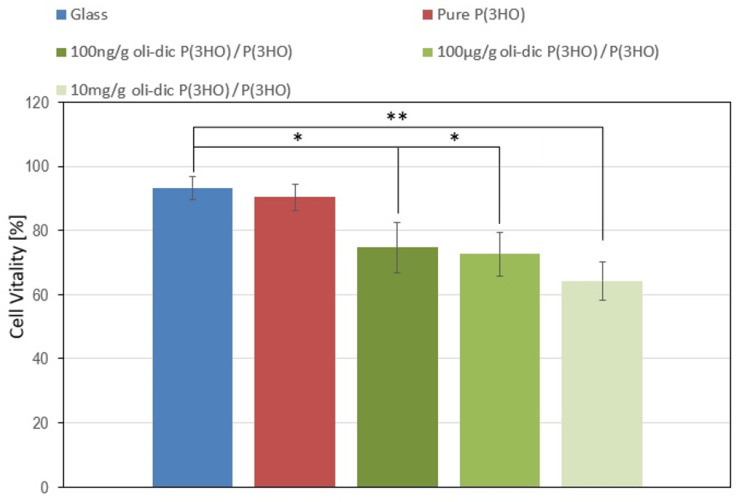
The cytotoxic assessment of P(3HO) enriched with different concentrations of diclofenac-modified oligomers (oli-dicP(3HO)) on MEF3T3 cells. Glass substrate was used as negative control. The results are statistically significant, where * *p* < 0.05, ** *p* < 0.001.

Selecting the appropriate concentration of an active substance that does not adversely affect the surrounding wound environment is a very important step in designing new transdermal wound dressings. Therefore, the standard double-staining vitality test was applied to assess the influence of diclofenac oligomers on fibroblast cells (MEF 3T3). The results were compared with those obtained for glass and pure P(3HO) substrates. According to ISO 10993-5, a reduction in cell vitality of more than 30% indicates that the material is cytotoxic [31]. The data show that the presence of diclofenac-modified conjugates causes a decrease in vitality rate by 15% for 100 ng/g concentration and continuously grows with increasing drug concentration (Figure 5). Al-Nimer et al. presented a similar effect of diclofenac on primary rat embryo fibroblast. They determined that the application of 625 µg of diclofenac did not induce a cytotoxic effect. However, cell viability was significantly reduced (74.9 ± 10.8%) [44].

According to the above observations, it is necessary to use the minimum concentrations of diclofenac-modified conjugates, given the therapeutic effects. Based on the results of this study, 100 µg/g of oli-dicP(3HO)/P(3HO) was selected as a safe concentration to be used in the construction of the presented wound dressings.

### 2.4. MEF3T3 Cells’ Behaviour on the Surface of Prepared Materials

**Figure 6:**
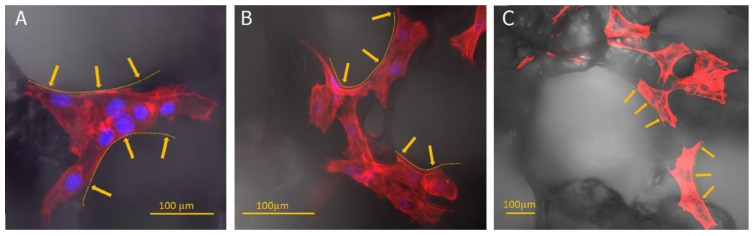
Microscopic images showing cells’ arrangement along the edges of the pores (orange dashed lines and arrows—placed inside the pore). The actin cytoskeleton is visualised in red (**A**–**C**) and cell nuclei (**A**,**B**) in blue. Panels A and B present images captured under 40× objective, panel C was imaged using a smaller (10×) magnification to exhibit a group of cells localised on the substrate.

**Figure 7:**
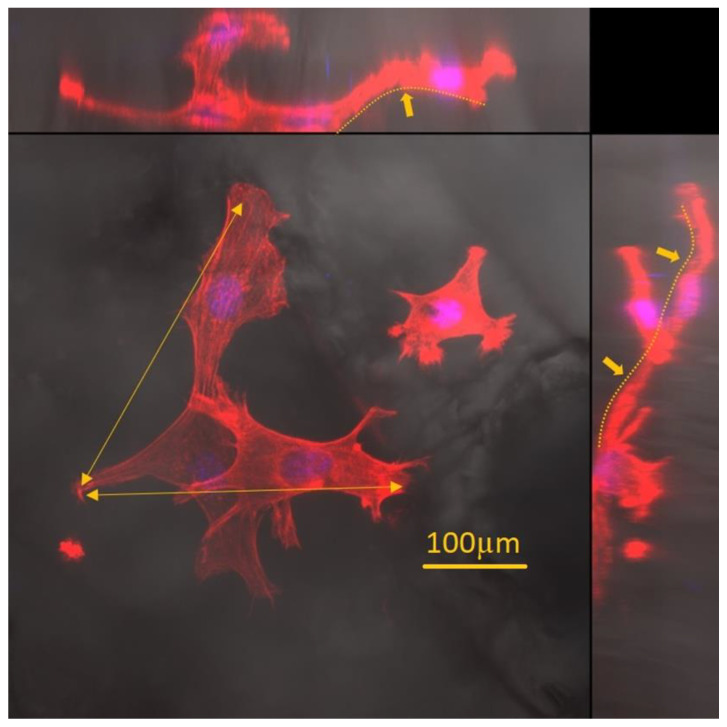
Three-dimensional image reconstruction in orthogonal projection shows cells’ tendency to overgrow pores and penetrate the 3D structure of the material. Some cells form protrusions and stretch out at the edges of the pore (orange arrow). The shape of the substrate is marked with a dashed line, which shows that the cells penetrate all irregularities and pores in the material. Actin filaments are presented in red, and the cell nucleus is in blue.

**Figure 8:**
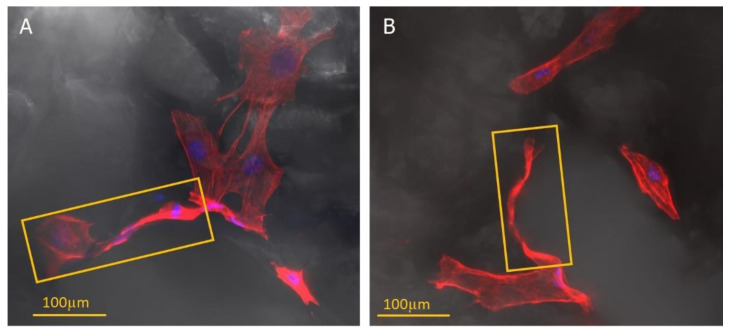
The images of the stained actin skeleton (red) and nuclei (blue) show the tendency of fibroblasts to form elongated protrusions that line the edge of the pore (marked with orange rectangles).

**Figure 9:**
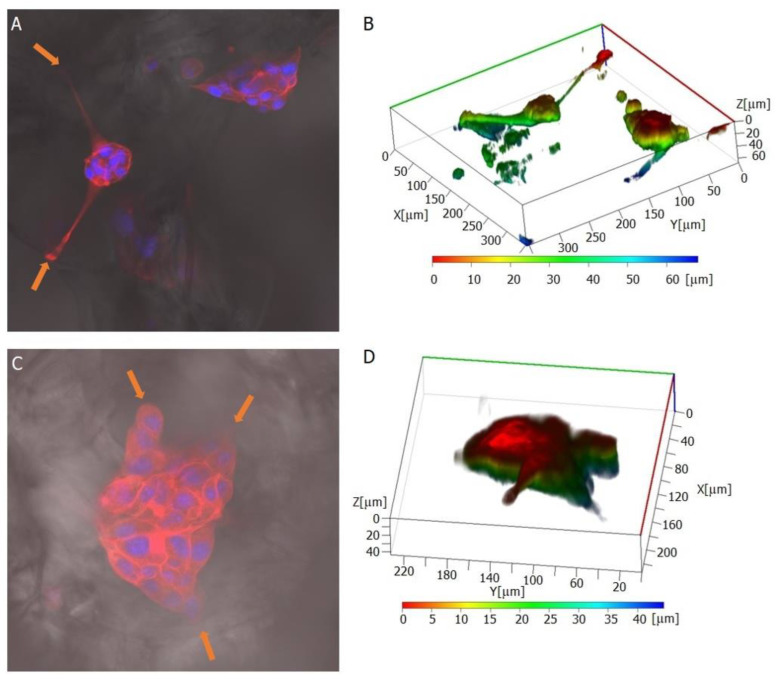
Cluster-like cell structures imaged in fluorescent mode (**A**,**C**) and 3D image reconstructions showing the spatial distribution of cell bodies (**B**,**D**). Cell attachment points at the pore edges are marked with orange arrows.

A confocal microscope imaged the fixed fibroblast cells on porous patches. The objects of observation were actin fibres, the nuclei, and the prepared materials’ structure. Three-dimensional reconstructions made it possible to analyse the arrangement of cells and their elements (e.g., filopodia) in the structures created inside the material. Cells’ tendency to align along the edge of the pores (Figure 6 and Figure 7) was observed. Additionally, the cells had a more elongated, spindle-like, and branched shape, and stress fibres were less visible (Figure 8). This observation is consistent with previous literature reports [45,46]. Moreover, physical parameters such as substrate rigidity and topography are essential for cell behaviour [29,47]. Research shows significant differences in the morphology of cells grown on 1D, 2D, and 3D substrates. It is assumed that cells feel the multidimensionality of their environment [48], and significant differences in cytoskeleton morphology can be found between 2D environments, where actin filaments are visible and organised, and 3D environments, in which the number and visibility of fibres are reduced. Actin is pushed to the Lateran borders of the cell extensions [49]. Another phenomenon observed was cluster-like structures created by the cells inside the pores. Fibroblast cells spread filopodia on the edge of the material, allowing the built structure to hang over empty spaces in the substrate (Figure 9). A reduced tendency to form a monolayer was also noted in favour of this type of growth. Literature reports that the fibroblasts grown on porous scaffolds with pores with a diameter between 10 μm and 90 μm created a monolayer after nine days of culturing [50] which is completely different from their behaviour on flat surfaces [29]. Cells tend to occupy the space inside the material’s pores, overgrowing them, which may indicate that the type of scaffold presented in this work may be suitable for producing functionalised absorbable tissue regeneration dressings. Literature reports indicate considerable interest in this product and technology [45,51,52,53]. The additional use of biodegradable and biocompatible materials in their production makes it possible to produce a new generation of dressing materials. This new product not only will not require invasive removal from the patient’s body, but also, thanks to its functionalisation with anti-inflammatory drugs, it will not exert negative pressure by preventing induction, for example, of inflammatory processes at the site of implantation.

### 2.5. In Vivo Study

The scope of this work is an assessment of the wound-healing possibilities of the created poly(3-hydroxyoctanoate) (P(3HO)) and diclofenac-enriched poly(3-hydroxyoctanoate) (P(3HO)/oli-dicP(3HO)) patches. Diclofenac is a model substance that, thanks to its anti-inflammatory properties, can reduce inflammation in the wound [54]. Wound healing is a complex, multi-stage, and precisely regulated process, which can be divided into four main stages: haemostasis (blood clotting), inflammation, proliferation (migration), and remodelling [55]. The haemostasis phase starts immediately after injury and takes minutes to hours, depending on the size and depth of the wound. Shortly after that, the inflammatory phase begins. Immune system cells migrate to the damage, protect the body from pathogens, and debride the wound. This phase takes between 3 and 14 days. However, it is often indicated that it should be completed on day seven after the injury. The next stage is the proliferation phase. It starts around day 2–3 after injury and finishes around 10–14 days. The final stage of the healing process is the remodelling phase, which can take months or even years.

**Figure 10:**
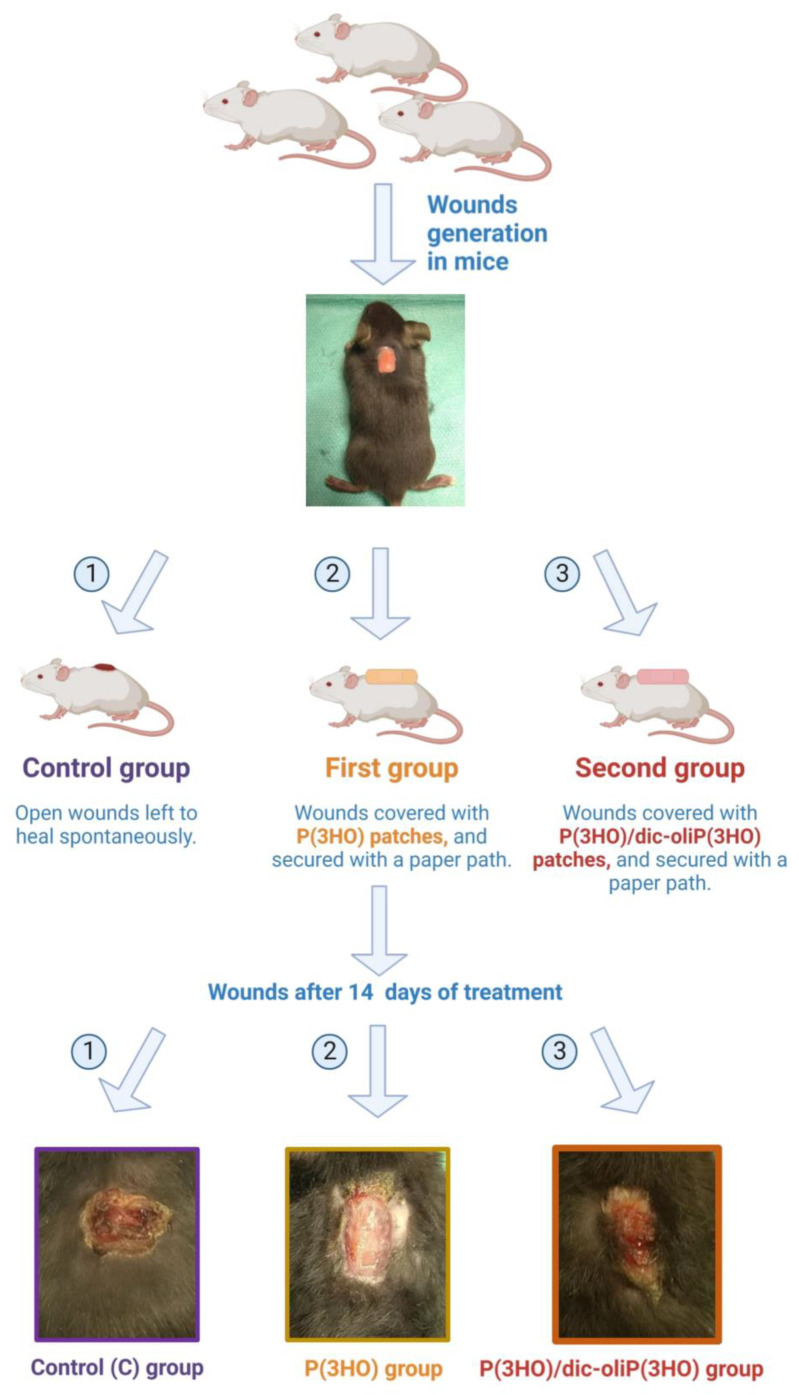
Schematic representation of the experimental procedure. In the beginning, rectangular (10 × 5 mm) wounds were created (photo at the top). The wound was left untreated (control group, C on the left side) or treated with unmodified (P(3HO)) or modified (P(3HO)/oli-dicP(3HO)) dressing (middle and right side, respectively). Created with BioRender.com.

Mice are the most widely used experimental model in wound-healing research. This is related to accessibility, as well as ease of maintenance, standardisation, and cheapness. Therefore, this model allows using a relatively high number of animals for statistic validation. In our study, 48 adult male mice of the C57BL/6 strain were divided into three study groups: (1) control (C); (2) P(3HO), and (3) P(3HO)/oli-dicP(3HO) (Figure 10). No complications related to the created wounds were observed in any study group (i.e., no weight loss, rapid/altered breathing (tachypnoea), loss of appetite, reduced physical activity, signs of marked pain or discomfort, unexpected vocalisation, wound exudation, etc.). Animals normally moved around the cage as well as ate and drank when they woke up after surgery.

The in vivo experiments were completed at two time points—after the 7th and 14th days. The first time point, the 7th day, was associated with the assessment of the inflammatory phase, whereas the second, the 14th day, corresponded to the time when the proliferation phase should occur. During the 7th (group I) and 14th days (group II) of the observation period, in the control group, wound contraction and scab (eschar) formation were observed. However, dressings placed in wounds and integrated with the injured area finally reduced wound contraction. It was also noticed that wounds with a P(3HO) and P(3HO)/oli-dicP(3HO) dressing were moister than the uncovered control. An ideal dressing should have some of the most important features: to maintain or provide adequate wound moisture and to ensure the possibility of gas exchange between wounded tissue and the environment [55]. One of the most important advantages of prepared dressings is that P(3HO) ensures moisture presence. At the same time, the pores visible in the dressing material allow for gas exchange and the removal of excess moisture (Figure 2A–F and Figure 3A–C). In both the unmodified dressings and those enriched with diclofenac, it was observed that the wounds were more moister than in the control group.

Due to the visible integration of the dressing materials within the tissues, wounds were not uncovered during the entire time the animals were observed. The dressing was removed from the wounds of two randomly selected mice—one from the P(3HO) and one from the P(3HO)/oli-dicP(3HO) group—to take a photograph. For all other mice, tissues were collected and analysed together with dressings.

**Figure 11:**
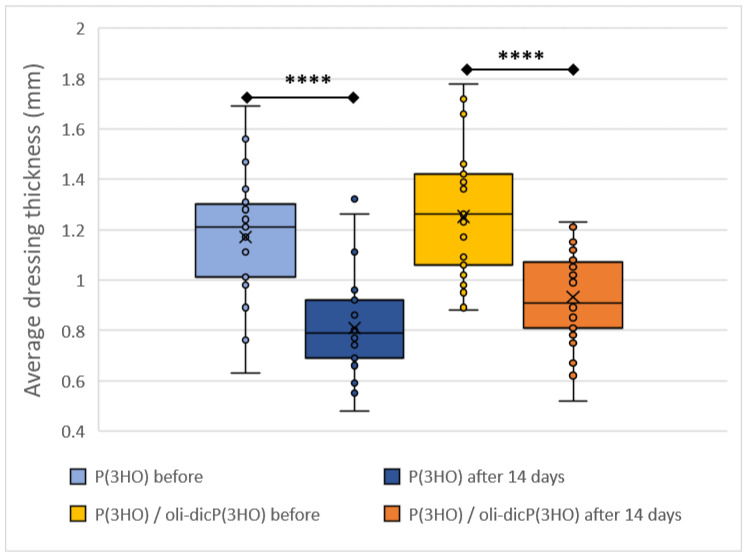
Comparison of average dressing thickness before operation and after 14 days of wound healing. Test: One-way ANOVA with post hoc Bonferroni’s multiple comparisons test, alpha 0.05 (95% confidence interval). Box plot showing the median (horizontal line); X (mean value). The box covers values from the first to the third quartile and the whiskers cover the total range. Significant digits (*) for *p*-value **** 0.0001.

When the dressings were removed, their physical appearances were changed. The patches were thinner and softer than before the wounds were treated. Therefore, the thickness of the dressings was measured before and after 14 days of treatment. Before applications, the thicknesses of P(3HO) and P(3HO)/oli-dicP(3HO) dressings were estimated to be 1.17 ± 0.23 mm and 1.25 ± 0.24 mm, respectively. Moreover, after the P(3HO) and P(3HO)/oli-dicP(3HO) patches were used, the thicknesses were 0.81 ± 0.20 mm and 0.93 ± 0.17 mm, respectively. To conclude, the thicknesses were reduced by 30.7% and 25.6%, respectively, for P(3HO) and P(3HO)/oli-dicP(3HO) (Figure 11). This is related to the bioresorption of P(3HO) and is very important for biomaterials used in regeneration processes. For proper regeneration, the scaffold must be replaced by natural tissue. Intermediate products of P(3HO) decomposition are 3-hydroxyacids (3HAs), natural metabolites produced in the liver in the β-oxidation process. Moreover, carboxylic acids are compounds described as agents that increase the penetration of the active ingredient (diclofenac) into the skin [54]. Furthermore, unnaturally occurring compounds were not introduced into the wound. Therefore, the absence of an inflammatory response at the site of biopolymer degradation was expected. In vivo experiments regarding the implementation of PHA-based scaffolds have been previously demonstrated. Volova et al. described in vivo studies on four types of polyhydroxyalkanoates (P3HB, P3HB/3HV, P3HB/3HHx, and P3HB/4HB), which were implemented into female Wistar rats for six months. Similarly to our study, no negative effects of the tested polymers on animals were found. No systemic inflammatory response, necroses, hematomas, granulomas, or pronounced swelling in the tissues surrounding the implants were observed. Instead, significant degradation of all examined scaffolds was estimated. This was most evident in the case of the scaffolds made of P3HB/3HHx and P3HB/3HB. After six months, their residual mass dropped to 10 and 20% of their initial mass. The most durable were matrices made of P3HB, which lost 55% of their initial mass [56].

Wound healing process was assessed using three methods: (1) immunohistochemistry (IHC), (2) gene expression analysis at the mRNA level determined using real-time PCR (qPCR), and (3) thanks to an immunoenzymatic method, ELISA (E), by examining serum levels of selected interleukins (ILs).

#### 2.5.1. Inflammation

The principal promise of the Hippocratic Oath is “primum non nocere”, which means “first, not harm”. Based on the above, the designed dressings should be harmless and not cause an inflammatory reaction. For this reason, on histological sections, some markers typical for mice skin γδ T cells (TCRγ/δ) and fibrocytes or monocytes (CD11b) were checked. Additionally, the levels of several cytokines and interleukins, both in the area of injury (mRNA level from the tissue covering the wound—*IL1A*, *IL6*, *IL10*, *IL18*, *TGFβ TSLP*, *ITGAM*, and *CCL20*) and systemically (serum levels of IL1A, IL6, and IL10) were determined.

**Figure 12:**
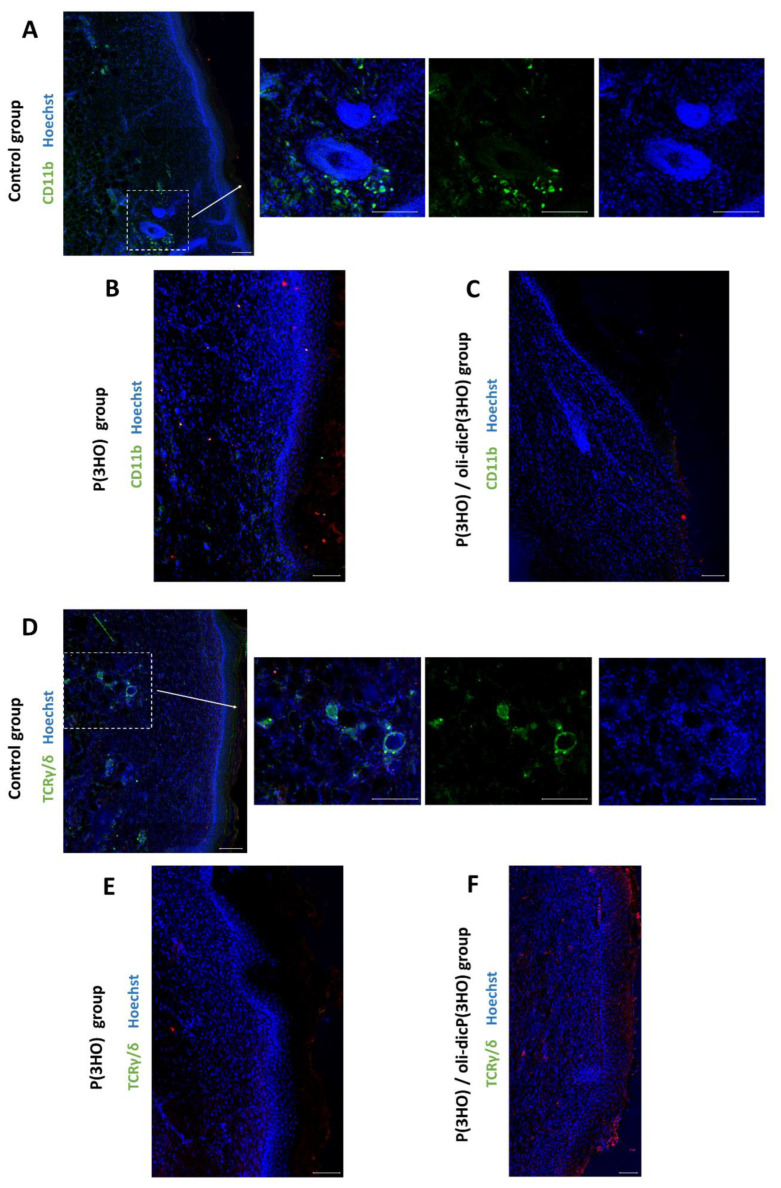
Confocal microscope photos show the presence of CD11b-positive cells (**A**–**C**) and mice skin γδ T cells (**D**–**F**) in the injured area. Scale bars—100 µm.

As can be seen in Figure 12, on day 14 of wound healing, in the injured area, only in the control group are positive CD11b (Figure 12A) and TCRγ/δ (Figure 12D) cells visible. For the wounds treated with P(3HO) and P(3HO)/oli-dicP(3HO) dressings, these cells were not observed (Figure 12B,C,E,F). These results confirm no infiltration of cells associated with developing inflammation in the examined tissues.

**Figure 13:**
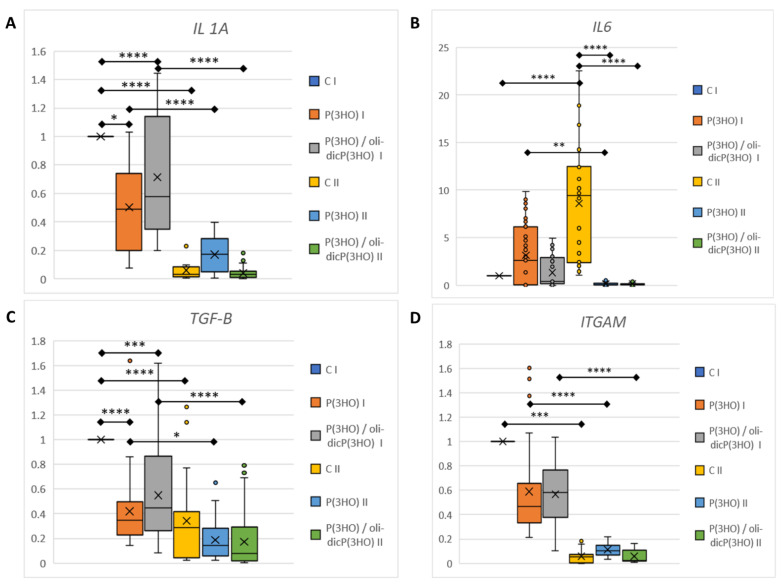
Relative gene expression of pro-inflammatory (*IL1*, *IL6*) (**A**,**B**) and anti-inflammatory cytokines (*TGF-β*) (**C**), as well as fibrocytes (*ITGAM*) (**D**), from the damaged tissue and the surrounding area on days 7 (I) and 14 (II). Test: One-way ANOVA with post hoc Bonferroni’s multiple comparisons test, alpha 0.05 (95% confidence interval), significant digits (*) for *p*-value * 0.0332, ** 0.0021, *** 0.0002, **** 0.0001. The plot shows the median (horizontal line), where X represents the mean value, the box covers values from the first to the third quartile, and whiskers represent the total range.

**Figure 14:**
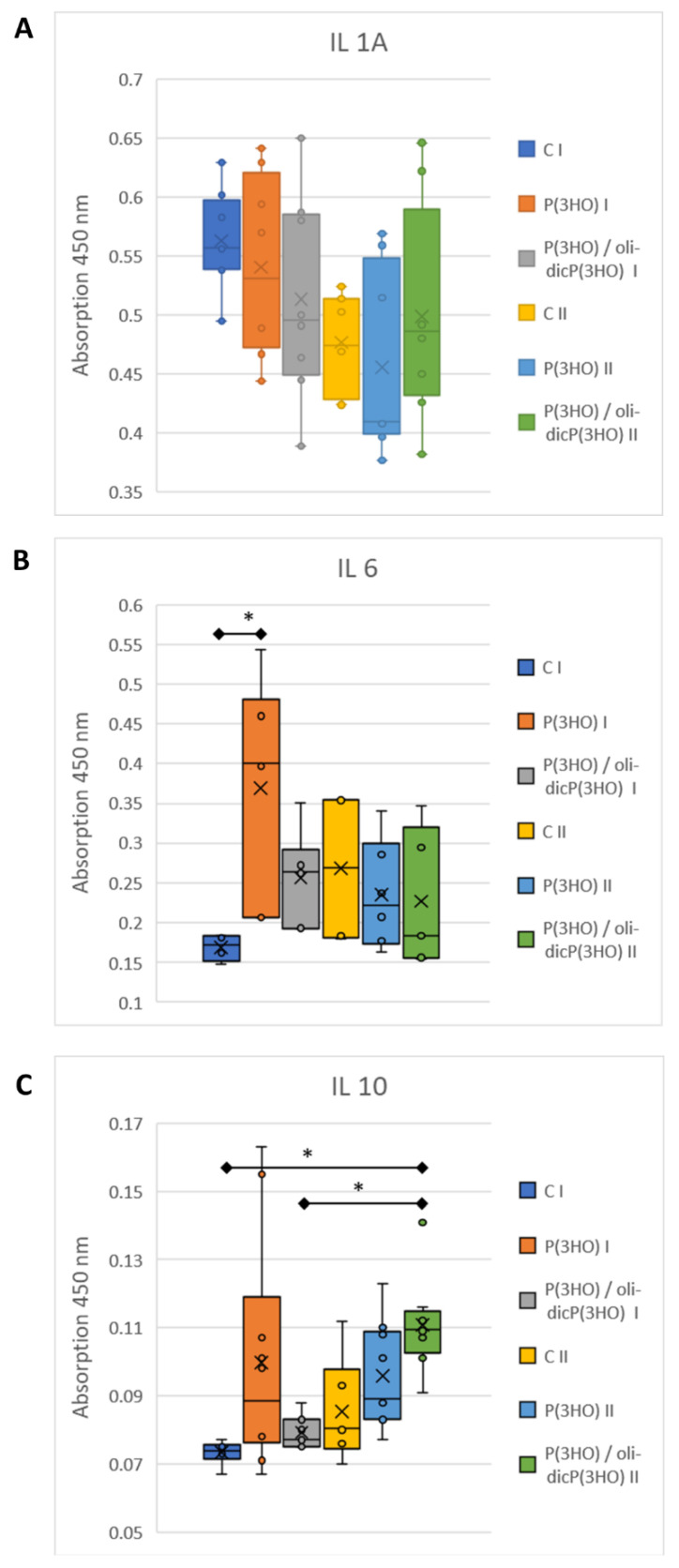
Average absorbance values of pro-inflammatory (IL1, IL6) (**A**,**B**) and anti-inflammatory cytokines (IL10) (**C**) in serum were obtained from mice’s peripheral blood on days 7 (I) and 14 (II). Test: One-way ANOVA with post hoc Bonferroni’s multiple comparisons test, alpha 0.05 (95% confidence interval), significant digits (*) for *p*-value * 0.0332. The box plot shows the median (horizontal line), where X represents the mean value, the box covers values from the first to the third quartile, and the whiskers represent the total range.

The initial injury to the mouse activated innate defences, which manifest as inflammation, a host defence mechanism. Therefore, we have investigated several of the factors that drive this response:

1. IL1a is one of the essential proinflammatory cytokines activating innate inflammation after injury. IL18 is also a proinflammatory cytokine that stimulates NK cells to produce IFN-γ. It is known that skin keratinocytes, as a first defence line, contain, inter alia, preformed IL1α and IL18, which are released immediately after injury from dying cells [57]. In the case of an uninfected, properly healing wound, proinflammatory cytokine levels should drop over time. In our study, at the mRNA level, there was a significant decrease in proinflammatory *IL1a* levels in all study groups between day 7 and day 14 of wound healing (Figure 13A). In addition, a significantly lower expression of *IL1a* was observed in the animals from the groups treated with P(3HO) and P(3HO)/oli-dicP(3HO) dressings on day 7. Furthermore, it was shown that *IL18* expression was at a very low level (limit of detection), which was comparable for all groups. In peripheral blood, a slight (not statistically significant) decrease in IL1a levels between days 7 and 14 was observed in all tested groups (Figure 14A).
2. IL6 is a pleiotropic cytokine responsible for activating the acute inflammatory phase, haematopoiesis, and immune reactions in response to an infection or tissue injuries. Prolonged expression of IL6 is associated with chronic inflammation, and its increased level is detected in chronic inflammatory diseases. Moreover, it was found that IL6 stimulates fibroblasts to exhibit collagen and glycosaminoglycans (GAG) production, and the IL6/JAK2/STAT3 signalling pathway is related to keloid formation [58,59]. In the presented experiment, it was observed that on day 7, the level of *IL6* mRNA was comparable in all groups, but on day 14 increased significantly in the control group, at the same time decreasing and being at a very low level in the experimental groups (Figure 13B). Interestingly, on day 7, an increase in the IL6 level in peripheral blood was observed in the animals treated with P(3HO) compared to the control group. Still, on day fourteen, it decreased (Figure 14B).
3. CCL20 is a strong chemokine produced by keratinocytes, which plays a role in immune cells (e.g., murine skin γδ T cells) recruitment after skin injury. It was found that dermis-resident Vγ4 T cells, activated by the CCR6-CCL20 pathway, infiltrate into the epidermis and are a significant source of IL17A. IL17A enhances the production of IL1β and IL23, leading to an increase in local inflammation at the early stages of wound healing, and excessive activation of CCL20—IL17A-IL1/23 loop activation delays skin wound healing [60]. In the presented work, on both days of observation, days 7 and 14, the mRNA level of *CCL20* was below the detection level, suggesting that initial inflammation had been suppressed.
4. TSLP (thymic stromal lymphopoietin) is one of the cytokines expressed mainly by epithelial cells and keratinocytes in the skin, lung, and intestine and plays a vital role in the initiation and maintenance of the allergic immune response. Its expression may be induced by mechanical injury, microbes, and the inflammatory cytokines IL4, IL13, and TNFα [61]. TSLP promotes the maturation of Langerhans and myeloid cells associated with the skin’s immune system. Activation of Langerhans cells by TSLP induces their synthesis of pro-inflammatory cytokines (e.g., TNFα) and is a sign of developing inflammation. In the tested animals in the control and experimental groups, TSLP mRNA levels in the wound and adjacent tissue were under the detection level on days 7 and 14.
5. *ITGAM* (integrin alpha M) gene code protein CD11b. CD11b is one of the fibrocytes (peripheral blood fibroblast-like cells) and monocytes marker. Fibrocytes are unique cells that exhibit properties of macrophages as well as fibroblasts. Fibrocytes play a pivotal role in wound healing during inflammatory and proliferation phases using numerous mechanisms: wound debridement (by, among other things, acting as antigen-presenting cells or phagocytic activity), tissue regeneration (by producing cytokines, chemokines, and growth factors), ECM synthesis, and wound closure via α-SMA-mediated contraction or angiogenesis (FGF2, VEGF, and PDGF synthesis). During the sub-healing phase, fibrocytes differentiate into myofibroblasts, depositing high levels of ECM components and MMPs [62]. In the experiments conducted in all study groups on day 7, the comparable expression of the *ITGAM* gene was demonstrated (Figure 13D). It was also shown that this expression significantly decreased on day 14 of observation, which is probably related to the transition of fibrocytes from the pro-inflammatory variant into the myofibroblast. In addition, this result was confirmed by immunocytochemical analysis (Figure 12A–C), in which, on day 14, single CD11b-positive cells were present only in the control group.
6. IL10 is a crucial anti-inflammatory cytokine that suppresses an excessive host immune response to injury or bacterial infection. After the injury, IL10 is produced by keratinocytes. IL10 triggers a robust suppressive response in macrophages and neutrophils, mainly via the transcriptional inhibition of cytokines and chemokines. It plays an important role in sterile wound healing. More importantly, it was shown that IL10 reduced the gene expression of type I collagen, fibronectin, and upregulated decorin expression in human skin fibroblasts by the TGF-beta downregulation [63]. In our study, *IL10* mRNA levels were deficient, almost undetectable. Still, an increase was observed in protein levels in peripheral blood, especially on day 14 in the case of animals treated with P(3HO)/oli-dicP(3HO) dressings (Figure 14C). Moreover, there is an apparent suppression of *TGF-beta* expression at the mRNA level (Figure 13C), which aligns with the authors’ observations. It was also found that high IL10 and reduced IL6 levels play an essential role in scarless, regenerative healing typical for the foetus [64]. In the presented experiment, a slight increase in IL10 levels in the blood was observed, with a concomitant decrease in IL6 and TGF-beta expression at the mRNA level. This is consistent with the observed smaller scab in animals treated with P(3HO) and P(3HO)/oli-dicP(3HO) dressings.

The presented results indicate that the dressings used reduced inflammation in the wound area and that the intermediate products of P(3HO) breakdown did not induce any other inflammatory reaction.

#### 2.5.2. Angiogenesis and Remodelling

**Figure 15:**
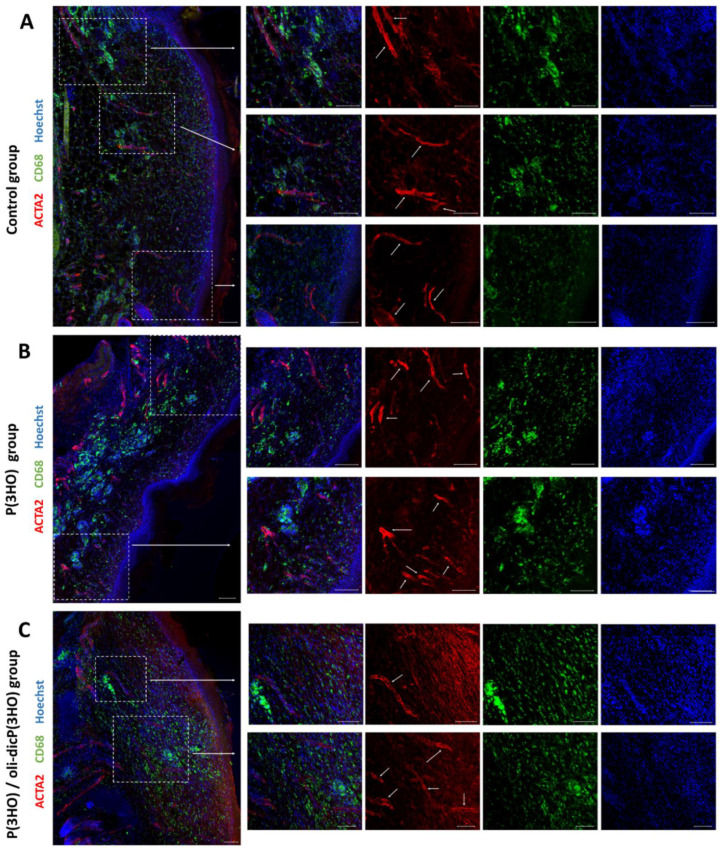
Confocal microscope photos show new blood vessels (ACTA2, red marked with white arrows) present in the healed area in control (**A**), P(3HO) (**B**) and P(3HO)/oli-dicP(3HO) (**C**) group and numerous CD68-positive cells (green). Scale bars—100 µm.

Another essential feature that a good dressing should have is the promotion of angiogenesis. In all experimental groups, it was possible to detect the formation of new blood vessels (ACTA2) in the healed areas (Figure 15 A–C). Other authors’ results also indicate that PHAs promote angiogenesis at damage sites. Gredes and colleagues evaluated the applicability of pure PHB patches in the regeneration of cranial defects in rats. They did not observe any visible or microscopic signs of inflammation or PHB rejection. After just 12 weeks post-implantation, they showed apparent bone regeneration and furthermore pronounced the development of blood vessels [65]. In other studies, Gumel et al. investigated the effect of poly (3-hydroxyalkanoates)-*co*-(6-hydroxyhexanoate) hydrogel on wound healing in rats. They found that wounds dressed with tested hydrogel showed extensively organised angiogenesis, evident collagen deposition, and enhanced fibrosis [66].

Another crucial step during wound healing is the removal of redundant cells and ECM elements and the remodelling of the newly developing ECM components. Results obtained in the immunohistochemistry showed that in wounds treated with dressings made of P(3HO) and P(3HO)/oli-dicP(3HO), a high number of CD68+ cells were present (Figure 15A–C). CD68 is a protein located in the lysosomal membrane and is expressed by different types of macrophages, monocytes, and osteoclasts. In mice, it is called macrosialin, the class D scavenger receptor. Interestingly, it is suggested that CD68+ cells are not associated with pathogen binding or innate, inflammatory, or humoral immune responses [67]. Moreover, the CD68 molecule is expressed in osteoclast-specific bone-resident macrophages, which are involved in bone maintenance, repair, and remodelling [68]. It has been suggested that CD68+ macrophages are involved in the clearance of regenerated tissue through the antigen presentation of apoptotic cells. It is also known that in humans, an increased CD68+ macrophage number results in increased secretion of angiogenic factors and is related to higher vascularity and metastasis [69]. In summary, they are an essential element in the remodelling phase.

**Figure 16:**
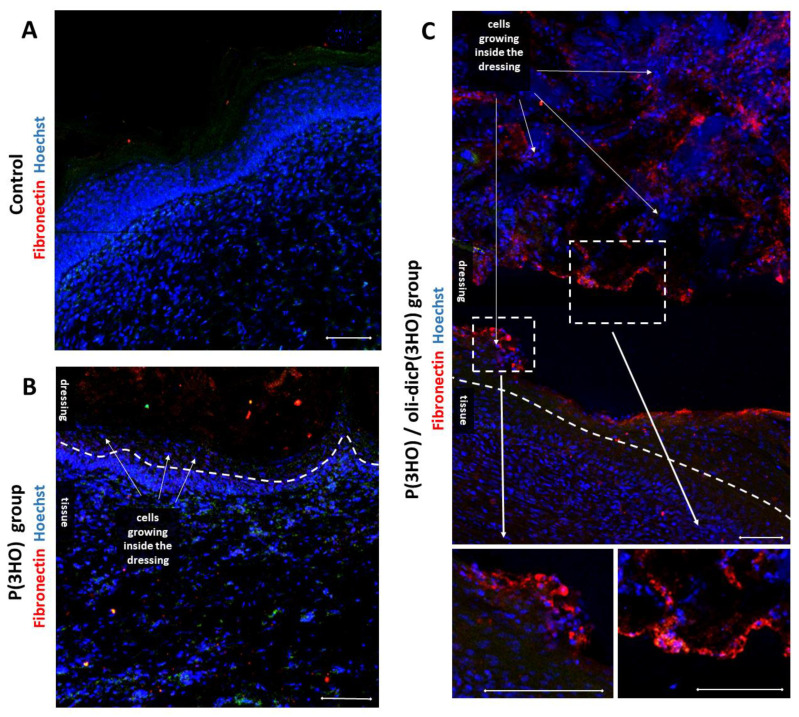
Confocal microscope images showing cells growing inside dressings—cell nuclei (Hochest (blue))—marked with white arrows (**B**,**C**), as well as fibronectin deposition (red) present in the healed tissue and P(3HO)/oli-dicP(3HO) dressing (**C**). Control group on picture (**A**). Scale bars—50 µm.

Fibronectin is one of the most important extracellular matrix components synthesised during wound healing. Fibronectin plays an important role in ECM formation, as well as reepithelialisation, by interaction with different cell types, induction of cell adhesion, or migration. There are two forms of fibronectin. The first one is soluble fibronectin from plasma, which is important in the first phase of wound healing and is responsible for forming a fibrin clot and blocking the bleeding immediately after an injury. The second one is made by fibroblasts, endothelial cells, and keratinocytes and is a mature tissue fibronectin. It was found that fibronectin enables keratinocyte migration in the wound bed. The invaluable role of fibronectin in wound healing is described in the excellent review written by Lenselink [70]. Interestingly, in our research, we observed fibronectin deposition inside P(3HO)/oli-dicP(3HO) dressing [Figure 16C]. In particular, it was synthesised by cells lying at the edges of the two dressing layers which had delaminated. Another very important observation is that both investigated dressings types were colonised by cells (Figure 16B,C), which confirms the material’s biocompatibility and may explain the intense degradation process manifested by the decreasing thickness of the dressing.

**Figure 17:**
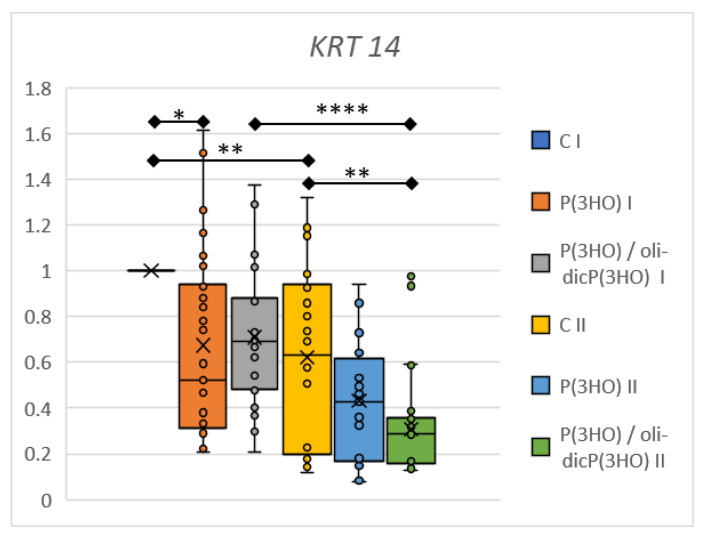
Relative gene expression of *Krt14* from the damaged tissue and the surrounding area on days 7 (I) and 14 (II). Test: One-way ANOVA with post hoc Bonferroni’s multiple comparisons test, alpha 0.05 (95% confidence interval), Significant digits (*) for *p*-value * 0.0332, ** 0.0021, **** 0.0001. Box plot showing the median (horizontal line), where X shows the mean value, the box covers values from the first to the third quartile, and the whiskers represents total range.

The expression of *Krt14* gene coding cytokeratin 14—a marker of highly proliferative keratinocyte stem cells localised to the basal layer of the epidermis—was also checked. These cells divide and move outward toward the skin surface/wound edge after injury. While moving toward the skin surface, they differentiated and lost their Krt14 expression [71]. From the evaluation of *Krt14* expression at the mRNA level, we can conclude that its levels decrease over time, especially in the control and P(3HO)/oli-dicP(3HO) group (Figure 17). This is reasonable, because in the initial stage of wound healing, new keratinocytes are needed, and after 14 days, they migrate, differentiate, and lose *Krt14* expression. Interestingly, a lower expression of the *Krt14* gene in the P(3HO) group (on day 7) and the P(3HO)/oli-dicP(3HO) group (on day 14) compared to the control was observed.

## 3. Materials and Methods

### 3.1. Materials Preparation with Physicochemical and Mechanical Characterisation Procedures

Poly(3-hydroxyoctanoate) (P(3HO)) was obtained in a high-cell-density cultivation process in a 5 L fed-batch reactor, according to Guzik et al. [72]. As the carbon source, octanoic acid (Sigma Aldrich, Poznań, Poland) was used. After the fermentation, the broth was centrifugated. Next, the cells were frozen and lyophilised. Further, the dried bacterial cells were resuspended in ethyl acetate (Chempur, Piekary Śląskie, Poland), and the polymer was isolated from the cells. P(3HO) was purified using activated charcoal (Carl Roth GmbH, Karlsruhe, Germany) and filtrated using 0.2 µm filters. After evaporation of the solvent, P(3HO) was precipitated using cooled methanol (Chempur, Piekary Śląskie, Poland), dried at 50 °C for 3 days, and then conditioned at 4 °C for at least a week.

The oligomers modified with diclofenac (Dic-oliP(3HO)) were obtained in a single-stage, solvent-free synthesis using a modified protocol described by Haraźna et al. [31]. The appropriate amounts of reagents (5 g of P(3HO) polymer and 0.75 g of *p*-toluenesulfonic acid (*p*-TSA) (Sigma Aldrich, Poznań, Poland)) were introduced into a round-bottom flask. When the molten state of P(3HO) and *p*-TSA were observed, 2 g of diclofenac (Fluorochem, Hadfield, UK) was introduced into a vessel. The reaction was performed using mechanical stirring for 2 min at 125 °C. Next, the reaction mixture was cooled and dissolved in dichloromethane (Avantor Chemicals, Gdańsk, Poland). The bottom (organic) layer was washed several times with 1 M sodium chloride aqueous solution (POCH, Gliwice, Poland) and water. After solvent evaporation, the mixture was dissolved in hexane (Sigma Aldrich, Poznań, Poland). Then, the appropriate amount of 2 M sodium hydroxide aqueous solution (POCH, Gliwice, Poland) was added and mixed on a magnetic stirrer for 30 min at room temperature. The mixture was washed with 1 M sodium chloride aqueous solution and water several times until a neutral pH was obtained. After that, hexane was evaporated under reduced pressure. The mixture was placed in a vacuum oven for 3 days at 45 °C. The reaction was performed in triplicate.

The porous P(3HO) and P(3HO)/oli-dic-P(3HO) foams were prepared by solvent casting/particulate leaching technique (SCPL). P(3HO) was resuspended in ethyl acetate (Chempur, Piekary Śląskie, Poland) (3% *w*/*v*). Sodium chloride (POCH, Gliwice, Poland) was ground and sieved to sizes of 100–300 µm. After that, NaCl was added to the polymer solution at a 95:5 NaCl/P(3HO) *w*/*w* ratio. The mixture was stirred overnight at room temperature. In the case of oligo-dic-P(3HO) foams, 175 µg of Dic-oliP(3HO) was added, and the mixture was stirred for 2 h at room temperature. Next, the mixture was poured into an appropriate dish and left to evaporate for 14 days. Next, the resulting composite was washed with deionised water to remove the sodium chloride grains. During the porogen leaching, the water was changed daily. The whole porogen leaching stage lasted no longer than one week. The water that remained in the foam was removed during the freeze-drying process. The foams were produced at least in triplicate.

The ^1^H NMR spectrum was obtained using a 300 Hz spectrometer (Bruker BioSpin GMbH, Rheinstetten, Germany) according to the protocol described by Haraźna et al. [31] The amount of attached diclofenac was determined using the equation

(1) $$\frac{m_{\mathrm{Dic}}\cdot n_{P\left(3\mathrm{HO}\right)}}{m_{P\left(3\mathrm{HO}\right)}\cdot m_{P\left(3\mathrm{HO}\right)}}=\frac{I_{\mathrm{Dic}}\;\cdot N_{P\left(3\mathrm{HO}\right)}}{I_{P\left(3\mathrm{HO}\right)}\;\cdot N_{\mathrm{Dic}}}$$

where m is the mass, n is the molarity, N is the number of nuclei giving rise to the signal, and I is integral. The MestRenova (Mastrelab Research, Santiago de Compostela, Spain) was used for all performed calculations and visualisation.

The GPC analysis was performed using a modified protocol described by Sofińska et al. [24]. In this order, the Phenogel 5u10E4A 300 × 7.8 mm and Phenogel 5u10E3A 300 × 7.8 mm columns connected in tandem (Phenomenex, Torrance, CA, USA) in a Shimadzu Prominance system (Shimadzu, Kyoto, Japan) with a UV–visible detector set at 240 nm were used. As an eluent, spectroscopic grade tetrahydrofuran (Chemsolve, Łódź, Poland) at a flow rate of 0.8 mL min^−1^ was used. The sample concentration was 30 g L^−1^. As standards, the polystyrene samples with different dispersity were used. All calculations were performed in triplicate.

The microstructure of obtained foams was investigated using scanning electron microscopy (Phenom Pure G5, PhenomWorld, Eindhoven, The Netherlands). All samples were previously sputtered with a gold layer.

Sample visualization and quantitative μCT analysis were performed using μCT (Bruker SkyScan 1172 micro-CT scanner, Bruker, Massachusetts, USA) according to paper published by Cichoń et al. [25]. Scanning parameters were adjusted so that the signal-to-noise ratio is optimal. The X-ray energy was 80 keV in combination with 0.5 mm Al filter were applied. The pixel size for all scans was 5 μm. A modified Feldkamp algorithm built in Nrecon software (Bruker Micro-CT, Bruker, MA, USA) was applied for image reconstruction procedure. All samples were visualised using CTVox package (Bruker Micro-CT), and a 3D volume rendering method with an optimised transfer function was used to obtain final images.

Tensile testing was conducted using an Inspekt Table Blue with a 10 kN load cell (Hegewald und Peschke MPT GmbH, Nossen, Germany) at room temperature. The measurements were performed using foams samples 35 mm in length, 5 mm in width, and 5–35 mm in thickness. The gauge length of the sample holder was 20 mm. A deformation rate of 20 mm per minute was applied. The stress–strain curve was used to determine Young’s modulus, tensile strength, and elongation at break. At least six measurements were made for each tested sample.

### 3.2. In Vitro Characterisation of Prepared Materials

For cell culturing and sample preparation, after the standard passage procedure, the immortal cell line of mouse embryonic fibroblasts (MEF 3T3) was grown for two days on the P(3HO) soft patches. Those culturing substrates were prepared and cast according to the protocol presented by Witko et al. [27]. The cells on the biopolymer substrates were grown in the incubator (Thermo Fisher Scientific, Waltham, MA, USA) under constant temperature (37 °C) and carbon dioxide concentration (5%). The medium used for culturing was DMEM (Dulbecco’s Modified Eagle Medium; Sigma Aldrich, Poznań, Poland) supplemented with 10% *v/v* of bovine serum (FBS; Sigma Aldrich, Poznań, Poland), and 1% *w/v* of antibiotics (penicillin and streptomycin; Sigma Aldrich, Poznań, Poland).

For cytotoxicity assessment cytotoxicity tests were made to estimate the cells’ vitality in the flat P(3HO) substrates, enriched with diclofenac oligomers. Fluorescent dyes were used for this experiment’s double staining method with Fluorescein Diacetate and Propidium Iodide (FDA/PI; Thermo Fisher Scientific, MA, USA). After the staining, samples were observed with the ×10 magnification lens. Laser wavelengths of 488 and 561 nm were used to excite the fluorescent stains. Final cell viability was estimated from the tile scan microscopic images, with an area around 200 mm^2^. The cell counting and statistical analysis were performed according to the protocol described by Witko et al. [29].

For cell staining and microscopic imaging, fibroblast cells were fixed, the actin cytoskeleton was stained with rhodamine-labelled phalloidin (Sigma Aldrich, Poznań, Poland), and the nuclei were dyed with DAPI (Thermo Fisher Scientific Waltham, MA, USA). The microscopic measurements were performed on Zeiss Axio Observer Z.1 with LSM 710 confocal module (Carl Zeiss NTS GmbH, Jena, Germany). Confocal Z- stacks were collected using an oil immersion objective with a magnification of 40× and NA of 1.4. The cells were observed by the confocal module. P(3HO) patches’ are visualised by a transmitted light channel (TPM). The image collection, analysis, and three-dimensional reconstructions were performed using Zeiss ZEN Black version 8,1,0,484 (Carl Zeiss NTS GmbH, Jena, Germany).

### 3.3. In Vivo Study

#### 3.3.1. Animals

The study was conducted using 48 adult male mice of the C57BL/6 strain, obtained from the Central Laboratory of Experimental Animals of Warsaw Medical University. Approval for the experiments was obtained from the 1st Local Ethical Committee for animal experiments (Act No. 170/2016, 16 November 2016, Warsaw). One mouse (from the second experimental group (P(3HO)/oli-dicP(3HO)) died on day six after surgery for unknown reasons (no visible complications, wound infection, etc.). Thus, 47 individuals ultimately participated in the experiment.

The experiments involved creating and dressing wounds and next observing the healing process. Two time variants of the experiment were used; in half of the animals, the healing process was followed for 7 (I) and the other half for 14 days (II). In each time-variant, animals were randomly divided into three groups of 8 individuals: (1) control (C) (spontaneously healing wounds, without dressing), (2) mice treated with unmodified biopolymer—P(3HO), and (3) mice treated with biopolymer modified with diclofenac (P(3HO)/oli-dicP(3HO)).

In all groups, animals were subjected to similar procedures. One day before surgery, the animals received paracetamol (200 mg/day) in drinking water. Initially, the animals were subjected to anaesthesia by intraperitoneal injection of ketamine (70 mg/kg of body weight) with xylazine (5 mg/kg of body weight). Then, after the upper part of the back was shaved, a piece of skin measuring 5 × 10 mm was cut off. The wound was covered with dressings in the experimental groups, while the control group was left untreated. After surgery, the animals were injected with metamizole sodium (Biovetalgin, 15 mg/kg of body weight). Then, for 7 or 14 days, the general condition of the animals was assessed, and the wound-healing process was observed. Next, animals were sacrificed, and the blood and tissue samples were collected. Fragments of skin containing the wound with adjacent tissues (about 2 mm on each side) were excised. Half of the collected tissue was frozen (−80 °C) and intended for mRNA isolation. The other half was fixed with 4% paraformaldehyde (PFA), dehydrated in the sucrose series (10, 20, and 30%), and used for immunohistochemical analysis.

#### 3.3.2. mRNA Isolation and Gene Expression Analysis

The mRNA isolation was performed by the method of Chomczyński/Sacchi by TRIZol-mediated extraction (acid guanidinium thiocyanate-phenol-chloroform) with minor modifications. Since TRIzol is a P(3HO) solvent, removing the layer containing the biopolymer from the tissue was necessary. Otherwise, it caused mRNA contamination that prevented further analysis. These dressings were used to measure the thickness of the material after 14 days of recovery.

The collected, frozen tissues were taken out of the ice and cut into smaller pieces using a scalpel. Then, the fragmented tissue was transferred into tubes and homogenised with TRIzol Reagent (Thermo Fisher Scientific Waltham, MA, USA). The homogenised mixture was left for 15 min at room temperature. Then, chloroform was added to the sample and shaken thoroughly, and samples were centrifuged (15 min at 4 °C, 12,000 rpm). The colourless aqueous phase (upper layer) was transferred to new tubes and mixed with isopropanol. Samples were incubated for 10 min at room temperature and centrifuged (10 min at 4 °C, 12,000 rpm). The supernatant was discarded, and the pellet was resuspended in 75% ethanol. Samples were centrifuged (5 min at 4 °C, 7500 rpm), and the supernatants were removed. The purification with ethanol was repeated twice. Then, the pellets were suspended in Diethyl prytocarbonate (DEPC)-treated water (Thermo Fisher Scientific Waltham, MA, USA). The RNA quality and concentration were estimated using a SmartSpec Plus Spectrophotometer (Bio-Rad Laboratories, Hercules, CA, USA).

The isolated mRNAs to evaluate gene expression with multiplex real-time PCR reactions. The reaction was performed according to the Applied Biosystems protocols using the TaqMan^®^ RNA-to-Ct™ 1-Step Kit (Thermo Fisher Scientific, Waltham, MA, USA) and suitable assays listed in Table 2. The GAPDH gene was used as a housekeeping gene. Amplification was performed in a ViiA 7 Real-Time PCR System (Applied Biosystems, Thermo Fisher Scientific, Waltham, MA, USA)).

#### 3.3.3. Immunohistochemistry

The isolated tissues were dehydrated in the sucrose series, fixed in paraformaldehyde, and then frozen at −20 °C. Before slicing, the tissues were covered with Leica OTC Tissue Freezing Medium (Leica Biosystems, Illinois, USA) and were left in the cryostat until freezing (at least half an hour). Then, tissues were cut into 20–30 µm thick slices using Leica Cryostat (Leica Biosystems, Illinois, USA). The tissue sections on the slides were either stained immediately or stored in a freezer (−20 °C).

Staining procedure: additional fixation of sections on slides (4% PFA, 15 min, RT), PBS rising, blocking with 5% Normal Goat Serum with 1% BSA, and permeabilisation with Triton ×100 (Sigma Aldrich, Merck, Germany) (1 h, RT), PBS rising, primary antibody (listed in Table 3) (1 h, RT), PBS rising, secondary antibody (1 h, RT, darkness), PBS rising, Hoechst 33342 (Thermo Fisher Scientific, Waltham, MA, USA) nuclei staining (15 min, RT, darkness), thorough PBS rinsing, and coverslips mounting with Dako fluorescent mounting medium (Dako north america, Carpinteria, CA, USA). Prepared slides were stored in a fridge (4 °C). Tissue sections were visualised with LSM 710 nlo confocal microscope (Zeiss, Jena, Germany).

#### 3.3.4. The Enzyme-Linked Immunosorbent Assay (ELISA)

The ELISA measured the peripheral blood’s interleukin (IL1, IL6 and IL10) level. The tests were carried out according to the manufacturer’s protocols. Three kits were used: IL-1 alpha Mouse ELISA Kit (ThermoFisher, Cat No. BMS611), IL-6 Mouse ELISA Kit (ThermoFisher, Cat No. KMC0061), and IL-10 Mouse ELISA Kit (ThermoFisher, Cat No. BMS614).

### 3.4. Statistical Analysis

The statistical analysis was performed using a one-way analysis of variance (ANOVA). The results were presented as mean values ± standard error (SE). The differences were considered to be statistically significant at: * *p* < 0.05; ** *p* < 0.01; *** *p* < 0.001 (Origin Pro 2019 Software, Origin Lab Corporation, Northampton, MA, USA).

## 4. Conclusions

The presented materials might be potential candidates as excellent dressings for wound treatment. Due to their hydrophobic properties, our dressings can reduce the risk of infection, e.g., when dipping a wounded limb in water in which pathogens may be present or with sweat, which, together with the exfoliated epithelium, is an excellent breeding ground for bacteria. Moreover, the investigated dressings reduce the expression of pro-inflammatory factors but do not completely inhibit the inflammatory phase, thus reducing the risk of chronic inflammation. Additionally, those patches meet requirements for modern dressings, such as maintenance of adequate wound moisture, appropriate gas exchange, angiogenesis promotion, and no wound irritation or induction of an inflammatory response. Prepared materials are soft, easy to handle and sterilise, and allow easy cutting to the shape and size of the wound. Complex, long-healing injuries (e.g., burns) can act as a protective barrier, usually provided by the skin. Moreover, the presented dressings are biocompatible and biodegradable and offer an ideal environment for the migration/growth of endogenous cells. These dressings can integrate with healed tissue and slowly biodegrade, and normal tissue will be reconstituted in its place.

## Figures and Tables

**Table 1:** Gel chromatography analysis of investigated samples.

	Mn	Mw	Dispersity Index
P(3HO) [24]	73.0	137.0	1.88
Oli-dicP(3HO)	6.19 ± 0.00	12.36 ± 0.16	2.00 ± 0.02

**Table 2:** Assays used in experiments, all from ThermoFisher.

Gene Name	Assay ID:	Catalogue Number	Dye Label
*Krt14*	Mm00516876_m1	4331182	FAM-MGB
*Itgam*	Mm00434455_m1	4331182	FAM-MGB
*Pecam1*	Mm01242576_m1	4331182	FAM-MGB
*Ccl20*	Mm01268754_m1	4331182	FAM-MGB
*Tslp*	Mm01157588_m1	4331182	FAM-MGB
*Il1a*	Mm00439620_m1	4331182	FAM-MGB
*Il6*	Mm00446190_m1	4331182	FAM-MGB
*Il10*	Mm01288386_m1	4331182	FAM-MGB
*Il18*	Mm00434225_m1	4331182	FAM-MGB
*Tgfb1*	Mm01178820_m1	4331182	FAM-MGB
*Gapdh*	Mm99999915_g1	4448490	VIC-MGB

**Table 3:** Primary and secondary antibodies.

Protein Name	Producent	Catalogue Number
	**Primary Antibodies**	
Krt14	Invitrogen/ThermoFisher	MA5-11599
CD11B	Invitrogen/ThermoFisher	14-0112-82
CD68	Invitrogen/ThermoFisher	14-0681-82
ACTA2	Proteintech	14395-1-AP
Fibronectin	Chemicon	AB2033
TCR gamma/delta	Invitrogen/ThermoFisher	14-5711-85
	**Secondary antibodies**	
Goat anti-Rabbit IgG (H+L) 488	Invitrogen/ThermoFisher	A32731
Goat anti-Rat IgG (H+L) 488	Invitrogen/ThermoFisher	A48262
Goat anti-Hamster IgG (H+L) 488	Invitrogen/ThermoFisher	A-21110
Goat anti-Rabbit IgG (H+L) 555	Invitrogen/ThermoFisher	A32732
Goat anti-Mouse IgG (H+L) 555	Invitrogen/ThermoFisher	A32723

## Data Availability

The data presented in this study are available on request from the corresponding author.
